# Improvements in cholesterol efflux capacity of HDL after bariatric surgery in patients with obesity: a meta-analysis

**DOI:** 10.1186/s12893-025-03233-9

**Published:** 2025-10-10

**Authors:** Tannaz Jamialahmadi, Elaheh Mirhadi, Željko Reiner, Saheem Ahmad, Bodor Bin Sheeha, Safia Obaidur Raba, Wael Almahmeed, Amirhossein Sahebkar

**Affiliations:** 1 Pharmaceutical Research Center, Pharmaceutical Technology Institute, Mashhad University of Medical Sciences, Mashhad, Iran; 2 Medical Toxicology Research Center, Mashhad University of Medical Sciences, Mashhad, Iran; 3 Biotechnology Research Center, Pharmaceutical Technology Institute, School of Pharmacy, Mashhad University of Medical Sciences, Mashhad, Iran; 4 Department of Internal Diseases, University Hospital Center Zagreb, Zagreb, Croatia; 5 Department of Medical Laboratory Sciences, College of Applied Medical Sciences, University of Hail, Hail, 2440 Saudi Arabia; 6 Department of Rehabilitation Sciences, College of Health and Rehabilitation Sciences, Princess Nourah Bint Abdulrahman University, P.O. Box 84428, Riyadh, 11671 Saudi Arabia; 7 Central Labs, King Khalid University, P.O. Box 960, AlQura’a, Abha Saudi Arabia; 8 Department of Clinical Laboratory Sciences, College of Applied Medical Sciences, King Khalid University, Abha, Saudi Arabia; 9 Heart and Vascular Institute, Cleveland Clinic Abu Dhabi, Abu Dhabi, United Arab Emirates; 10 Biotechnology Research Center, Pharmaceutical Technology Institute, Mashhad University of Medical Sciences, Mashhad, Iran; 11 Centre for Research Impact & Outcome, Chitkara College of Pharmacy, Chitkara University, Rajpura, Punjab 140401,, India; 12 Applied Biomedical Research Center, Basic Sciences Research Institute, Mashhad University of Medical Sciences, Mashhad, Iran

**Keywords:** Bariatric surgery, HDL cholesterol efflux capacity, Cardiovascular disease risk, Coronary heart disease

## Abstract

**Background:**

Bariatric surgery can decrease cardiovascular risk because of its effects on atherosclerotic cardiovascular disease (ASCVD) risk factors such as dyslipidemia, diabetes mellitus, and hypertension. It has been hypothesized that lipoprotein biomarkers related to low-density lipoproteins (LDL) and high-density lipoproteins (HDL) as well as their subfractions and macrophage cholesterol efflux capacity (CEC) might be important in cardiovascular risk assessment.

**Objective:**

The aim of this systematic review and meta-analysis was to evaluate the CEC of HDL as a negative risk factor for ASCVD in individuals with obesity following bariatric surgery.

**Data source:**

PubMed, Scopus, Web of Science and Scholar were searched from inception to May, 2024.

**Studies selection:**

Clinical studies that reported CEC data with a follow-up after bariatric surgery were eligible for meta-analysis.

**Data extraction:**

Two independent reviewers extracted data and assessed the risk of bias.

**Results:**

Among the 55 articles identified, 8 studies measuring cholesterol efflux capacity (CEC) after bariatric surgery were analyzed. Four studies indicated a nonsignificant increase in total CEC after Roux-en-Y gastric bypass (RYGB) or sleeve gastrectomy (SG) (SMD: 0.361, *p* = 0.256). Five studies showed a significant reduction in ABCA1-dependent CEC after the surgery (SMD: -1.044, 95% CI: -1.916, -0.173, *p* = 0.019). In contrast, five studies reported a significant increase in ABCA1-independent CEC (SMD: 0.932, 95% CI: 0.142, 1.722, *p* = 0.021). Subgroup analyses showed that total CEC varied by type of surgery, with significant increases for SG (SMD: 0.748, *p* < 0.001) but not for RYGB (SMD: -0.341, *p* = 0.650). ABCA1-dependent CEC decreased significantly after RYGB (SMD: -1.545, *p* = 0.015), while no significant changes were observed for ABCA1-independent CEC after both types of surgery.

**Conclusion:**

Bariatric surgery, particularly SG, is associated with clinically significant increase in total post-surgery CEC which might be beneficial concerning the ASCVD risk.

## Introduction

 We are witnessing a global pandemic of overweight and obesity with a devastating increasing trend. Obesity is a chronic metabolic disease which causes or is a risk factor for many other serious diseases. Among them, cardiovascular diseases (CVD) are the most important. Obesity is associated not only with increased atherosclerotic CVD (ASCVD) morbidity but also with increased ASCVD mortality, particularly coronary heart disease (CHD) morbidity and mortality [1]. However, obesity is also associated with arterial hypertension, dyslipidemia, metabolic syndrome (MetSy), type 2 diabetes mellitus (T2DM), metabolic dysfunction-associated steatotic liver disease (MASLD), chronic kidney disease, asthma, different types of cancers, gallstones, pancreatitis, psoriasis, atopic dermatitis and autoimmune arthritis [2–5]. For instance, obesity is second to smoking the next most preventable cause of death in the US and it is estimated that one in five deaths in Americans aged 40 to 85 years could be attributed to obesity [6].

As already mentioned, obesity is often associated with dyslipidemia and/or MetSy characterized by low concentration of HDL-cholesterol (HDL-C) which is, taken in general, a well-known risk factor for ASCVD [7]. Nevertheless, besides HDL-C concentration the capacity of cholesterol efflux to HDL particles is an essential indicator of the atherogenic/antiatherogenic potential. HDL particles play a crucial role in reverse cholesterol transport (RCT) thus having an important role in prevention of atherosclerotic plaque formation [8]. Namely, it has to be stressed that an inverse association exists between cholesterol efflux capacity (CEC) of HDL and risk for ASCVD which is independent of HDL-C levels [9]. CEC measures the ability of HDL particles to promote cholesterol efflux from macrophages which is the first step in reverse cholesterol transport. Since ATP-binding cassette transporter A1 (ABCA1) plays an essential role in formation of HDL it has to be mentioned also that very low HDL-C levels and defective CEC may be caused by defects in ABCA1 [10].

Weight loss in obese persons, no matter how achieved, decreases the risk of ASCVD, cardiovascular events and mortality. Bariatric surgery is a surgical treatment which is used primarily for extremely obese patients to decrease their excessive weight. Most often used types of bariatric surgery are sleeve gastrectomy (SG) and Roux-en-Y gastric bypass (RYGB) [11]. It has been shown that bariatric surgery decreases all-cause mortality, new-onset heart failure as well as risk for myocardial infarction and ischemic stroke [12, 13]. It also decreases intima-media thickness (IMT) which is a surrogate marker of early atherosclerosis and has a good correlation with atherosclerotic CHD [14]. This is due to the effects of bariatric surgery on ASCVD risk factors. It has been shown that bariatric surgery caused a decrease in total cholesterol, triglycerides, extremely atherogenic oxidized LDL particles and apolipoprotein B, and an increase in HDL-C and apolipoprotein A concentrations [15, 16]. It also decreases the concentrations of atherogenic lipoprotein (a) [Lp(a)] in the blood [17] A meta-analysis published 8 years ago indicated that bariatric surgery caused a significant increase of HDL-C at one year after the surgery [18]. A more recent meta-analysis also showed that bariatric surgery in severely obese patients causes a decrease of oxidized LDL particles [16]. This type of surgery improves also other risk factors for ASCVD including hypertension [19]. Nevertheless, all the metabolic changes caused by bariatric surgery and the exact mechanisms driving these changes remain poorly understood.

The aim of this study was to evaluate the changes in HDL-mediated CEC after two most often used types of bariatric surgery in patients with obesity since it has been hypothesized that decreased CEC could be a potential negative risk factor for ASCVD.

## Methods

### Data sources and searches

In accordance with the Preferred Reporting Items for Systematic Reviews and Meta-Analyses (PRISMA) guidelines, a search was conducted on PubMed, Scopus, Web of Science, and Google Scholar from their inception until May 20, 2024. The search utilized specific keywords in the titles and abstracts: (“metabolic surgery” OR “bariatric surgery” OR “gastric bypass” OR “Roux-en-y” OR OR “gastric band” OR “biliopancreatic diversion” OR gastrectom* OR “weight loss surgery” OR “weight-loss surgery” OR “gastrointestinal diversion” OR “duodenal switch” OR gastroenterostom* OR “obesity surgery” OR “jejunoileal bypass” OR “bariatric procedure” OR “sleeve surgery” OR gastroplast*) AND (“Cholesterol Efflux” OR “Cholesterol Efflux Regulatory Protein” OR “ATP Binding Cassette Sub Family A Member 1” OR “abca protein” OR “Cholesterol efflux capacity” OR “CEC” OR “apoa exchange rate” OR “AER”))

### Study selection

We included studies involving individuals with severe obesity who underwent Roux-en-Y Gastric Bypass (RYGB) or sleeve gastrectomy (SG), encompassing both randomized controlled trials and observational studies that provided data on cholesterol efflux capacity (CEC) before surgery and during follow-up.

### Data extraction and quality assessment

Two authors (EM, TJ) reviewed the full texts of the initially selected articles to verify whether they reported CEC data both prior to surgery and at follow-up. In cases of duplicate studies, the one with the largest sample size was chosen for inclusion.

### Quality assessment

The quality of the observational studies included in this meta-analysis was evaluated using the Newcastle-Ottawa Scale (NOS).

### Data synthesis and statistical analysis

Using Comprehensive Meta-Analysis (CMA) V4 software, we conducted random-effects modeling of standardized mean differences (SMD) based on cholesterol efflux and the type of surgical procedure. Heterogeneity was evaluated using I² statistics. Our main analysis focused on comparing the mean differences in CEC between pre-surgery values and post-surgery follow-up in patients who underwent RYGB or SG.

### Meta-regression

To assess the impact of changes in BMI post-surgery on effect size, a random-effects meta-regression model was employed, treating these variables as independent factors.

## Results

**Fig. 1:**
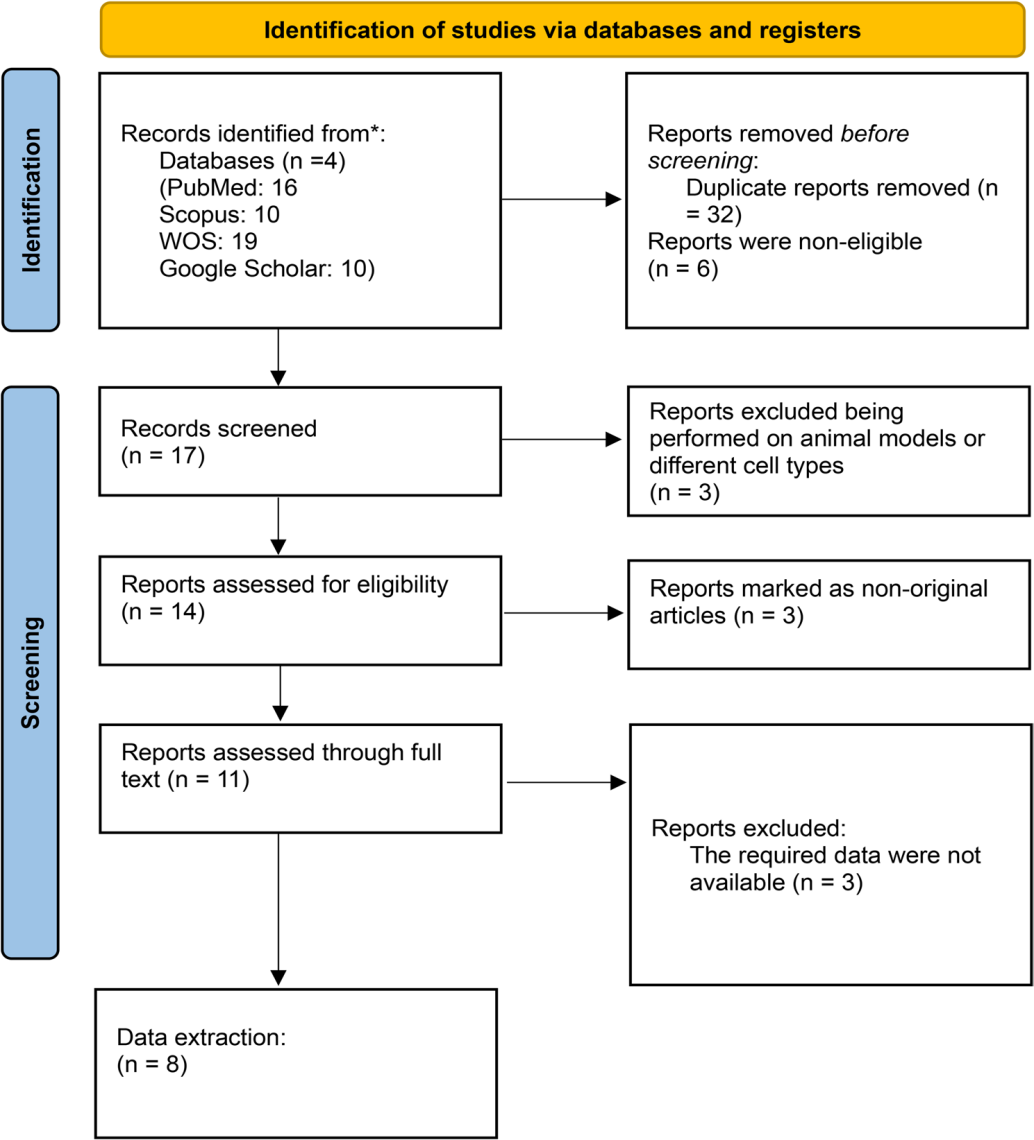
flowchart of included studies

Of the 55 articles identified from the database search, 32 were duplicated and 6 studies were non-eligible. Total of 17 met the inclusion criteria (Fig. 1). Of these, 9 studies were excluded for various reasons, including inadequate data reporting (*n* = 3), being animal models or done on different cell types (*n* = 3) or non-original articles (*n* = 3), leaving 8 studies that measured CEC following RYGB or SG for analysis. (Fig. 1). (Table 1)

**Table 1:** Demographic characteristics of the included studies

Study	Year	Study design	Target population	Duration of study	Type of surgery	*N*	Age (years)	Female, *N*. (%)	BMI (kg/m^2^)	Cholesterol Efflux (%)	Ref
Aron-Wisnewsky et al.	2011	Prospective clinical research	Morbidly obese women were recruited and followed up before and 6 months after RYGBP	6 months	Roux-en-Y bypass (RYGBP)	34	18–50	100%	44.3 [6]	ABCG1: 23.33 [0.62] SR-BI-dependent efflux: 20.38 [0.93] ABCA1-dependent efflux: 25.35 [0.78]	[20]
Davidson et al.	2017	Pilot study with a prospective biospecimen repository protocol	Severely obese adolescent males	12 months	Vertical sleeve gastrectomy	10	17.4	-	52.1 [9.8]	0.94 cholesterol efflux capacity increased by 12%	[21]
Heffron et al.	2018	A prospective study	Severely obese, non-diabetic, premenopausal Hispanic women not using lipid lowering medications	6 months	RYGB SG	RYGB: 31 SG: 36	RYGB: 30.4 SG: 34	100%	SYGB: 43.1 [5.7] SG: 45 [7]	RYGB: Global cholesterol efflux capacity (%): 8.3 [1.8], cAMP-inducible efflux capacity (%): 3.1 [1.2], nonABCA1-mediated efflux capacity (%): 5.2 [1.1] SG: Global cholesterol efflux capacity (%): 8.1 [2.5], cAMP-inducible efflux capacity (%): 3.4 [1.5], nonABCA1-mediated efflux capacity (%): 4.9 [0.9]	[22]
Kjellmo et al.	2018	A prospective study	Morbidly obese patients	12 months	Laparoscopic Roux-en-Y gastric bypass and Biliopancreatic diversion with duodenal switch	34	43.09	20 (59%)	44.8 [6.9]	35.62 [8.84]	[23]
Lorkowski et al.	2018	A prospective cohort study	Patients with obesity and type 2 diabetes	60 months	RYGB SG	RYGB: 37 SG: 33	RYGB: 48.52 SG: 48.54	RYGB: 26 (54.1%) SG: 27 (81.8%)	RYGB: 37.1 [3.32] SG: 35.87 [4.32]	ABCA1-independent cholesterol efflux capacity: RYGB: 0.96 [0.08] SG: 0.95 [0.08]	[24]
HO et al.	2021	A prospective clinical study	Severely obese patients	12 months	RYGB	53	48.9	40 (75%)	51.38 [9.12]	12.94 [3.79]	[25]
Thakkar et al.	2021	A longitudinal cohort study	Obese patients	3 months	SG RYGB	56	42.9	41 (73%)	45.8 [6.3]	SG: 0.89 [0.1] RYGB: 0.92 [0.14]	[26]
Adam et al.	2022	A prospective study	Obese patients	12 months	RYGB	44	49.5	32 (73%)	51.18 [9.05]	13.1 [3.76]	[27]

### Quality assessment of the studies

Table 2, presents the quality assessment of selected studies.

**Table 2:** Quality assessment of the included studies in accordance with the Newcastle-Ottawa scale

Study	Selection				Comparability†		Outcome				
	Representativeness of the Exposed Cohort	Selection of the Non-Exposed Cohort	Ascertainment of Exposure	Demonstration That Outcome of Interest Was Not Present at Start of Study	Comparability of Cohorts on the Basis of the Design or Analysis		Assessment of Outcome	Was Follow-Up Long Enough for Outcomes to Occur		Adequacy of Follow Up of Cohorts	
Aron-Wisnewsky 2011 [20]	*	-	*	*	-		*	*		*	
Davidson 2017 [21]	*	*	*	*	-		*	*		*	
Heffron 2018 [22]	*	*	*	*	-		*	*		*	
Kjellmo 2018 [23]	*	*	*	*	-		*	*		*	
Lorkowski 2018 [24]	*	*	*	*	-		*	*		*	
Ho 2021 [25]	*	*	*	*	-		*	*		*	
Thakkar 2021 [26]	*	*	*	*	-		*	*		*	
Adam 2022 [27]	*	*	*	*	*		*	*		*	

Effect of RYGB or SG on total cholesterol efflux capacity (CEC)

**Fig. 2:**
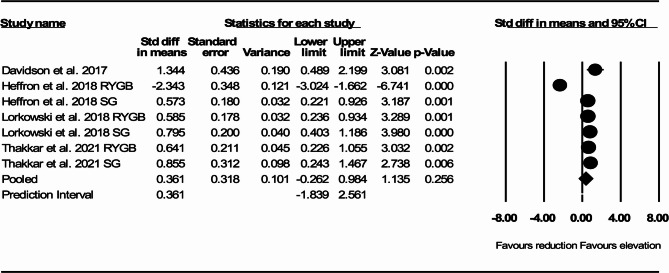
Forest plots representing standardized mean difference and 95% confidence intervals (CIs) for the effect of RYGB or SG on total cholesterol efflux capacity (CEC)

In 4 studies that reported total CEC before and after RYGB or SG, there was a nonsignificant increase in total CEC compared with pre-surgery measurement (SMD: 0.361, 95% CI: −0.262, 0.984, *p* = 0.256, I^2^:91.99, Q-value: 74.96) (Fig. 2).

### Effect of RYGB or SG on ABCA1 dependent CEC

**Fig. 3:**
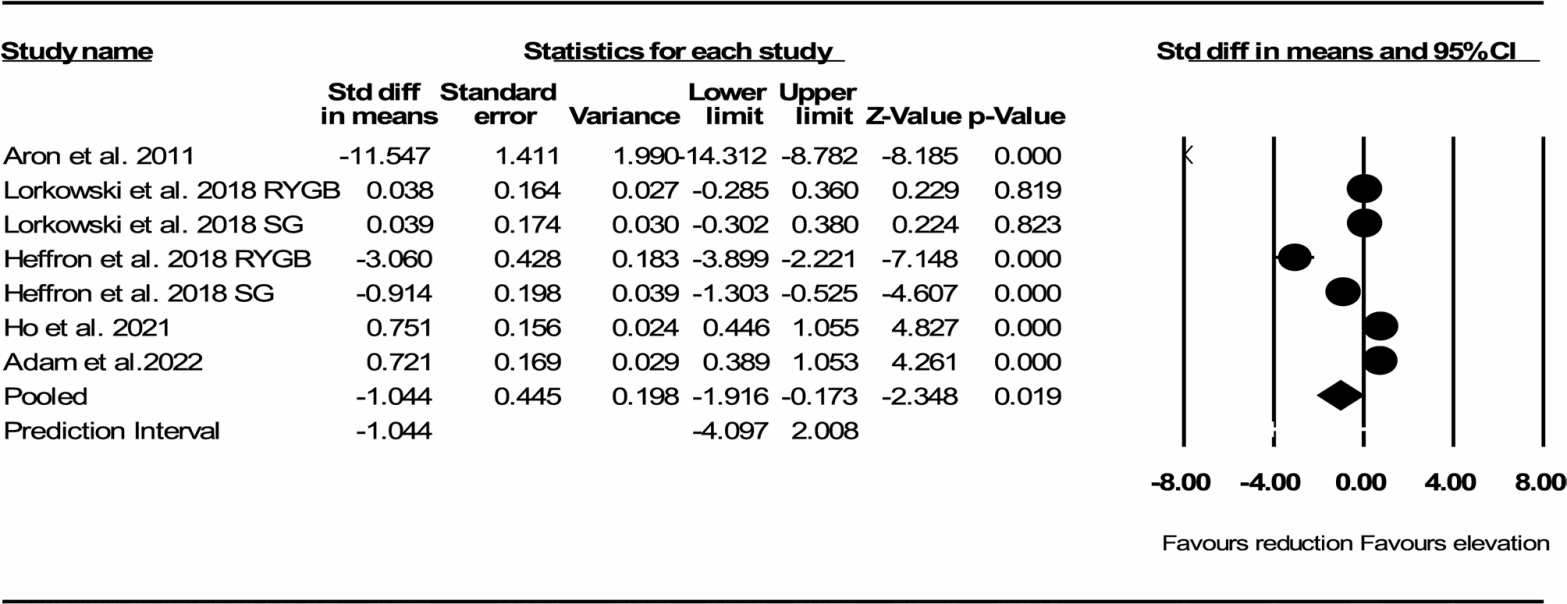
Forest plots representing standardized mean difference and 95% confidence intervals (CIs) for the effect of RYGB or SG on ABCA1 dependent cholesterol efflux capacity (CEC)

In 5 studies that reported ABCA1 dependent CEC before and after RYGB or SG there was a significant reduction in ABCA1 dependent CEC compared with pre-surgery measurement (SMD: −1.044, 95% CI: −1.916, −0.173, *p* = 0.019, I^2^:96.66, Q-value: 179.88) (Fig. 3).

### Effect of RYGB or SG on ABCA1 independent CEC

**Fig. 4:**
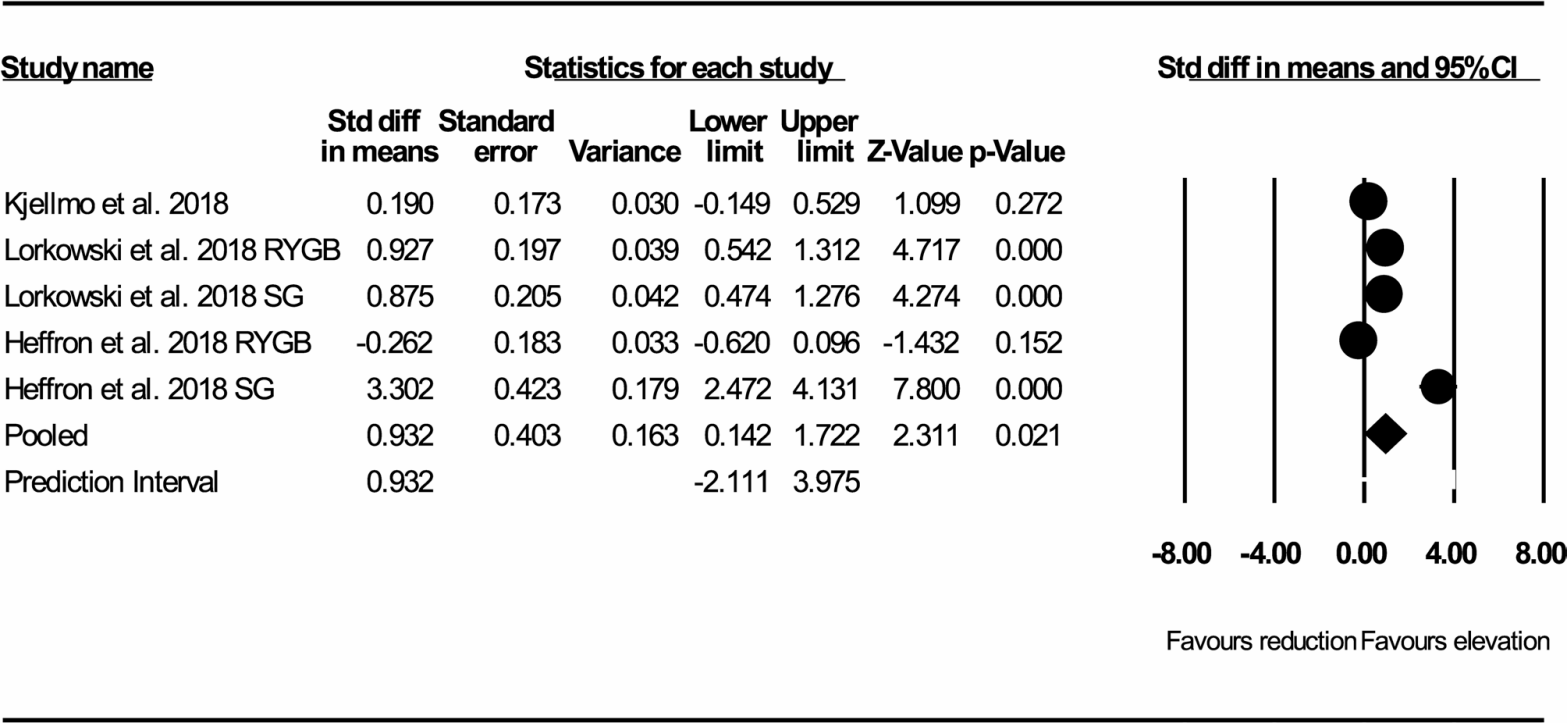
Forest plots representing standardized mean difference and 95% confidence intervals (CIs) for the effect of RYGB or SG on ABCA1 independent cholesterol efflux capacity (CEC)

In 5 studies that reported ABCA1 independent CEC before and after RYGB or SG there was a significant increase in ABCA1 independent CEC compared with pre-surgery measurement (SMD: 0.932, 95% CI: 0.142, 1.722, *p* = 0.021, I^2^:95.54, Q-value: 67.34) (Fig. 4).

### Subgroup analysis

A subgroup analysis was also performed based on the type of surgery. Bariatric surgery was associated with total CEC depending upon the type of surgery (SMD: 0.748, 95% CI: 0.516, 0.980, *p* < 0.001; for SG and SMD: −0.341, 95% CI: −1.815, 1.133, *p* = 0.650; for RYGB (Table 3).

**Table 3:** Subgroup analysis based on type of surgery

Subgroup analysis	SMD	Lower limit	Upper limit	*p*-value
Total CEC				
SG RYGB	0.748 −0.341	0.516 −1.815	0.980 1.133	< 0.001 0.650
ABCA1-dependent CEC				
SG RYGB	−0.433	−1.367	0.501	0.364
	−1.545	−2.792	−0.298	0.015
ABCA1-independent CEC				
SG RYGB	2.060	−0.317	4.438	0.089
	0.331	−0.834	1.496	0.578

Bariatric surgery was associated with ABCA1 dependent CEC depending upon the type of surgery (SMD: −0.433, 95% CI: −1.367, 0.501, *p* = 0.364; for SG and SMD: −1.545, 95% CI: −2.792, −0.298, *p* = 0.015; for RYGB).

Bariatric surgery was associated with ABCA1 independent CEC depending upon the type of surgery (SMD: 2.060, 95% CI: −0.317, 4.438, *p* = 0.089; for SG and SMD: 0.331, 95% CI: −0.834, 1.496, *p* = 0.578; for RYGB).

### Meta-regression

**Fig. 5:**
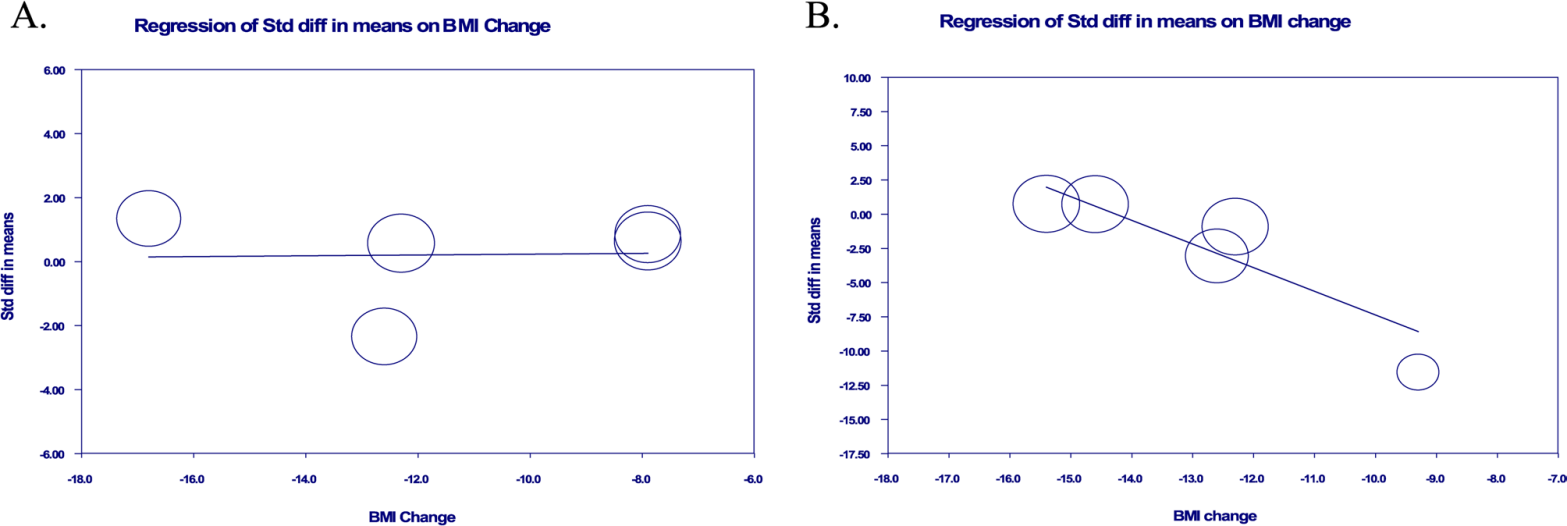
Random-effects meta-regression for evaluating the effect of (**A**): BMI change in total CEC, (**B**): BMI change in ABCA1 dependent CEC

The findings from the meta-regression analysis aimed at evaluating the relationship between alterations in BMI and changes in total CEC after surgery revealed no significant link. The slope was reported as 0.012, with a 95% confidence interval ranging from − 0.336 to 0.361, and the p-value was 0.944, indicating a lack of correlation. (Fig. 5A)

The findings from the meta-regression analysis aimed at evaluating the relationship between alterations in BMI and changes in ABCA1 dependent CEC after surgery revealed significant correlation. The slope was reported as −1.729, with a 95% confidence interval ranging from − 2.329 to 0.0775, and the p-value was − 1.130, p-value < 0.001. (Fig. 5B)

### Publication bias

#### Total cholesterol efflux capacity (CEC)

**Fig. 6:**
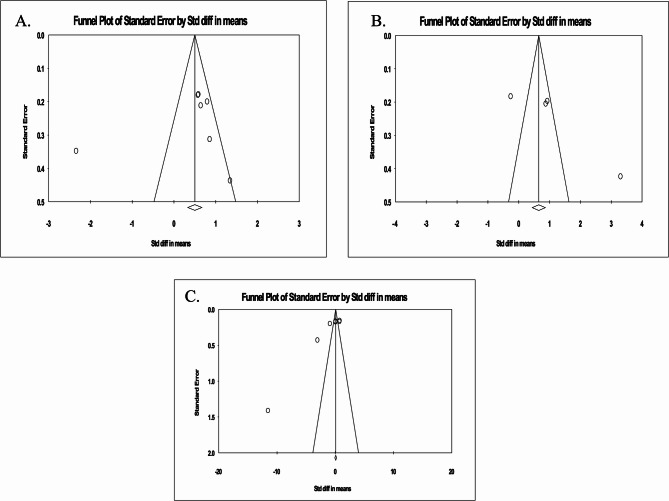
Funnel plot detailing publication bias in the studies which reported the effect of RYGB on (**A**) Total cholesterol efflux capacity (CEC), (**B**) ABCA1 independent CEC, (**C**) ABCA1 dependent CEC

As shown in Fig. 6A, the studies were assessed for publication bias using a funnel plot asymmetry test, with Egger’s test demonstrating no bias (intercept = −3.567, standard error = 4.801; 95% CI = −15.911, 8.775, t = 0.742, df = 5, two-tailed *p* = 0.490). Begg’s tests showing no bias (Kendall’s Tau with continuity correction = 0.190, z = 0.600, two-tailed *p*-value = 0.452) in detecting the impact of RYGB on circulating total cholesterol. The trim and fill test revealed two “missing” studies to adjust for bias, and the “fail-safe N” analysis showed that 35 papers could change the study’s conclusion.

#### ABCA1 dependent CEC

As shown in Fig. 6B, the studies were assessed for publication bias using a funnel plot asymmetry test, with Egger’s test demonstrating bias (intercept = −11.182, standard error = 2.537; 95% CI = −17.704, −4.660, t = 4.407, df = 5, two-tailed *p* = 0.006). Begg’s tests showed bias (Kendall’s Tau with continuity correction − 0.761, z = 2.403, two-tailed *p*-value = 0.016) in detecting the impact of bariatric surgery on LDL levels. The trim and fill test revealed no “missing” study to adjust for bias, and the “fail-safe N” analysis showed that 22 papers could change the study’s conclusion.

#### ABCA1 independent CEC

As shown in Fig. 6C, the studies were assessed for publication bias using a funnel plot asymmetry test, with Egger’s test demonstrating no bias (intercept = 13.423, standard error = 5.431; 95% CI = −9.948, 36.794, t = 2.471, df = 2, two-tailed *p* = 0.132). Begg’s tests showed no bias (Kendall’s Tau with continuity correction 0.500, z = 1.019, two-tailed *p*-value = 0.308) in detecting the impact of bariatric surgery on HDL levels. The trim and fill test revealed one “missing” study to adjust for bias, and the “fail-safe N” analysis showed that 58 papers could change the study’s conclusion.

## Discussion

The results of this meta-analysis showed an increase in total post-surgery CEC in patients who had SG, a significant reduction in ABCA1-dependent CEC after surgery in patients who had RYGB and no significant changes in ABCA1-independent CEC after both types of surgery.

An increase in CEC following SG suggests an altered HDL particles functionality after bariatric surgery which might be beneficial concerning the risk for ASCVD. This is important because of the data shown in a recent meta-analysis indicating that decreased CEC was strongly linearly correlated with an increased risk of CHD but not cardiovascular mortality, independently of HDL-C levels [28].

Another meta-analysis showed that decreased CEC was an independent risk factor for CHD and it could predict all-cause and cardiovascular mortality in patients with CHD [29]. In accordance with these results were the results of other two meta-analyses suggesting that higher CEC or improved HDL function, rather than higher HDL-C concentrations, were associated with lower adverse ASCVD outcomes [30, 31]. Therefore, it seems appropriate to state that HDL particles functionality rather than their number is important in atherogenesis/atheroprevention.

In obese patients, HDL particles tend to be smaller and more prone to oxidation, losing their ability to prevent LDL oxidation. Bariatric surgery not only increases plasma HDL cholesterol (HDLc) levels but also corrects these functional abnormalities. Previous studies have indicated that weight loss interventions, including diet and surgery, can enhance HDL particle size, shifting the distribution towards larger particles [15, 20–24, 32, 33].

Post-surgery, cholesterol esterification in HDL is a crucial factor that promotes the formation of larger HDL particles and improves their antioxidative properties. Correlation analyses reveal a positive relationship between HDL’s antioxidant capacity and the levels of esterified cholesterol. Conversely, there are negative correlations between HDL’s susceptibility to oxidation and apoA-I content—indicating that lower apoA-I levels are associated with shorter lag phases for oxidation—and between antioxidant capacity and HDL protein content [34].

Additionally, bariatric surgery normalizes the levels of certain apolipoproteins in HDL. HDL from obese patients typically has higher apoC-III and lower apoJ compared to controls, but these levels are normalized after surgery. This alteration is significant as similar changes in apoC-III and apoJ have been observed in HDL from patients with coronary artery disease [35].

It is important to stress that there is an important difference between ABCA1-dependent and ABCA1-indepencent CEC and the relationship of these two parameters with different types of HDL particles. Namely, it has been shown that ABCA1-dependent CEC is correlated with lipid-poor smaller HDL particles while ABCA1-independent CEC is correlated with lipid-rich larger HDL particles [36, 37]. Since it has been proven that obese subjects have significantly decreased larger and potentially more anti-atherogenic HDL particles compared to non-obese individuals [38], and it has been suggested that bariatric surgery increases the number of large HDL particles, and in these patients an increased ABCA1-independent CEC could be found [24], the results of our meta-analysis could not confirm this hypothesis.

There are some important clinical implications of this study. For instance, since it has been suggested that ABCA1-dependent CEC is reduced after RYGB, weight loss achieved after SG offers much better opportunity to prevent ASCVD and probably could account for the significant reduction in ASCVD mortality in obese patients after bariatric surgery as shown in a number of studies in which the effect of all the types of bariatric surgery on ASCVD was analyzed together. On the other hand, the anatomic rearrangement achieved by RYGB increases the concentration of circulating bile acids which reduce the expression of apolipoprotein AI, the main apolipoprotein of HDL particles, thus possibly having a negative effect on HDL particles formation [39].

Understanding CEC changes caused by different types of bariatric surgery can help to evaluate CVD risk reduction after the surgery thus suggesting the best approach in extremely obese patients who need ASCVD prevention. Anyhow, the results of this meta-analysis suggest the necessity for further research on HDL functionality after different types of bariatric surgery, and particularly the underlying mechanisms which affect HDL particles functionality after bariatric surgery.

This study has some limitations. One is a limited number of studies included and heterogeneity in study designs. The other is the variability in follow-up durations and surgical techniques used. The difference in included studies populations in terms of ethnicity might also be one of the limitations.

## Conclusion

Bariatric surgery, particularly SG, is associated with a clinically significant increase in total CEC following the procedure, which may have beneficial implications for reducing the risk of ASCVD. However, it is crucial to acknowledge the limitations of our findings, particularly the low statistical power of the study. In patients who underwent RYGB, there was a notable reduction in ABCA1-dependent CEC post-surgery, whereas no significant changes were observed in ABCA1-independent CEC for either surgical approach. Given the limited power of our analysis due to limited number of studies, we must consider caution in drawing strong causal or mechanistic conclusions from the current data.

## Data Availability

The data are available upon reasonable request from the **corresponding author: ** Amirhossein Sahebkar, amir_saheb2000@yahoo.com.

## Abbreviations

ABCA1: ATP-binding cassette transporter A1
ASCVD: Atherosclerotic cardiovascular disease
BMI: Body mass index
CEC: Cholesterol efflux capacity
CHD: Coronary heart disease
HDL: High-density lipoprotein
HDL-C: High-density lipoprotein cholesterol
LDL: Low-density lipoprotein
Lp(a): Lipoprotein (a)
MASLD: Metabolic dysfunction-associated steatotic liver disease
MetSy: Metabolic syndrome
NOS: Newcastle-ottawa scale
Q: Cochran’s Q statistic
RCT: Reverse cholesterol transport
RYGB: Roux-en-Y gastric bypass
SG: Sleeve gastrectomy
SMD: Standardized mean difference
T2DM: Type 2 diabetes mellitus
